# A Reliable Method to Assess Keel Bone Fractures in Laying Hens From Radiographs Using a Tagged Visual Analogue Scale

**DOI:** 10.3389/fvets.2018.00124

**Published:** 2018-06-07

**Authors:** Christina Rufener, Sarah Baur, Ariane Stratmann, Michael J. Toscano

**Affiliations:** ^1^Centre for Proper Housing of Poultry and Rabbits (ZTHZ), Animal Welfare Division, Veterinary Public Health Institute, University of Bern, Zollikofen, Switzerland; ^2^Clinical Radiology, Department of Clinical Veterinary Science, Vetsuisse Faculty, University of Bern, Bern, Switzerland

**Keywords:** keel bone fractures, radiograph, visual analogue scale, laying hens, reliability, scoring system

## Abstract

Up to 97% of laying hens housed in aviary systems are affected by keel bone fractures. Due to the scope of the problem, multiple efforts investigating causes and consequences of fractures have been conducted. The most frequently used techniques to detect fractures lack accuracy and provide only vague information (palpation) or cannot be conducted longitudinally (dissection). Radiographic imaging overcomes these weaknesses as it allows longitudinal observations and provides detailed information for individual fractures of which a single keel may have several at different locations and of different origins. However, no standardized system exists to assess fracture severity from radiographs if multiple fractures are present. The aim of this study was therefore to test the reliability of a scoring system assessing the aggregate severity of multiple fractures, taking into account the characteristics of all present fractures (e.g., locations, callus formation, width of fracture gaps). We developed a scoring system based on a tagged visual analogue scale, ranging from score 0 (no fracture) to score 5 (extremely severe) with intermediate tags for scores 1, 2, 3, and 4. A catalog of example scores was provided to describe the range of each score visually. An online tutorial with an introduction, training and scoring session was completed by 14 participants with varying experience involving laying hens and keel bone damage. For inter-observer reliability, we found an Intraclass correlation coefficient (ICC) of 0.985 with a 95% confidence interval of 0.974 < ICC < 0.993 (average-rating, absolute-agreement, two-way random-effects model). Intraclass correlation coefficient for intra-observer reliability was 0.923 with a 95% confidence interval of 0.879 < ICC < 0.951 (single-rating, absolute-agreement, two-way mixed-effects model). Intra-observer reliability ranged from 0.704 to 1.0 indicating excellent agreement and similar ratings across and within participants. Further, high ICCs suggest that the introduction and the training sessions provided were adequate tools to prepare observers for the assessment task despite various backgrounds of the participants. Nonetheless, the validity of this scoring system needs to be investigated further in order to link responses of interest and biological relevance with the specific severity values resulting from our scoring system.

## Introduction

Keel bone fractures in laying hens are an important welfare issue because of their likely association with pain and suffering (1–3). Due to the scope of the problem with reports of 97% of laying hens within a flock manifesting fractures (4), multiple research studies investigating prevalence, causes, risk factors, and consequences of keel bone fractures have been conducted in recent years. However, estimates of fracture prevalence under comparable conditions vary considerably. For instance, fracture prevalence in studies using multiple strains at 59–63 weeks of age in an aviary housing system ranged from 11.6 (5) to 97.0% (4). Besides the effect of management factors (e.g., feeding), different methods of fracture assessment could contribute to the large range of reported fracture prevalence. To assess keel bone fractures in live hens, palpation is the most commonly used method due to high throughput and low cost, but comprehensive training is crucial (6). Palpation can also be conducted longitudinally but lacks sensitivity to specific fracture characteristics. Dissection could provide much more detail such as fracture number and location (7), but has the obvious disadvantage that it can only be performed on hens after death and therefore does not allow for longitudinal observations.

In human medicine, the severity of fractures is scored according to the fracture location, morphological characteristics such as the complexity of the fracture or fragment displacement, difficulty of treatment and prognosis (8, 9). In order to assess these measures on keel bones, various diagnostic imaging techniques have been examined in laying hens, e.g., ultrasonography (10), CT scans (11, 12), or radiographic imaging (13, 14). Radiographic imaging is sensitive for fracture numbers and characteristics and facilitates the detection of fractures at the dorsal site of the keel (14). Furthermore, radiographs allow longitudinal keel bone fracture assessment and provide images that can be evaluated repeatedly. Although radiography equipment is expensive and requires training, radiographic imaging could be carried out on farm (15).

Radiography offers several benefits over other techniques (e.g., palpation) to study keel bone fractures as they allow longitudinal, on-farm observations in combination with the opportunity for detailed assessment of fracture severity similarly or better than visual inspection after dissection. Although no standardized severity scoring system for radiographs of keel bones exists, a standardized system to assess fracture severity would be important as it is not clear if there is a threshold where accumulated damage results in impaired hen welfare. There is evidence that hens suffering from keel bone fractures experience pain (16–18) but it is not known how the severity of fractures affects pain intensity. For instance, mild damage might be within the coping capacity of the hen and only have minimal, short-term effects on welfare (14). In order to investigate the magnitude of a response to fractures, a continuous variable for fracture severity would be preferable over a categorical variable (i.e., presence or absence) due to various functional and statistical reasons (19). Further, the quantification of aggregate damage of individual hens is required as hens often suffer from multiple fractures. Therefore, the aim of this study was to test the reliability of a radiograph scoring system based on a tagged visual analogue scale taking into account multiple fractures of an individual hen and their characteristics (e.g., number, location, type, callus formation).

## Animals, materials and methods

### Radiographic procedure

The scoring system was based on radiographic images from another ongoing study in our research group (FSVO; project number 2.15.05). Based on the design of the main study, keel bones of 150 aviary-housed hens (75 Lohmann Selected Leghorn, 75 Lohmann Brown) were radiographed at 11 time points throughout the laying cycle (22, 25, 28, 33, 37, 40, 45, 49, 54, 57, and 61 weeks of age). One latero-lateral image was produced per hen per time point. From this pre-existing set of 1,622 radiographs, specific images were selected for the current reliability trial according to the criteria described below.

Hens were radiographed with a mobile radiograph unit (GIERTH HF 200 ML; radiograph tube Toshiba D-124 with maximal acceleration voltage of 100 kV; radiograph plate Canon CXDI-50G; software Canon CXDI Control Software NE) using a distance of 80 cm and voltage of 46 kV/2.4 mAs. To induce immobility during the radiographic procedure, hens were hung upside down in metal shackles fixed by a wooden frame according to the protocol described by Širovnik and Toscano (15). As inversion was shown to induce fearfulness (20), hens were handled carefully within the shortest timeframe possible, resulting in approximately 10–20 s of inversion per hen and radiograph. The pressure of shackles on feet and legs could cause pain (21), thus shackles were padded with foam material and no pressure was applied to fix the hen's feet in the shackles. To insert the legs into the metal slots of the shackle, both legs of the hen were held in one hand and the hen's body was stabilized with the other hand to prevent defensive movements which could increase the risk for bone damage (22). Results of the main study demonstrated that repeatedly radiographed hens (11 radiographs within 41 weeks) did not show higher fracture prevalence than hens radiographed only once (data not shown).

Radiographs were imported to the PACS (Picture Archiving and Communication System; IMPAX EE, Agfa Healthcare, Bonn, Germany) of the Department of Clinical Radiology (Vetsuisse Faculty, University of Bern) as DICOM files. For analysis, radiographs were downloaded from the PACS as JPEG files.

### Radiograph scoring system

Fracture numbers and characteristics (e.g., location, type) varied among age and hybrids, thus the set of 1,622 radiographic images represented a broad and externally valid range of keel bone fractures. As most hens were affected by multiple fractures (2.8 ± 1.7 fractures per hen, ranging from 0 to 9 fractures per hen), the aggregate severity of all present fractures in one keel bone at each time point was defined as the total amount of bone affected by fractures of an individual hen. Due to the complexity of keel damage in consequence of multiple fractures, specific fracture characteristics such as the number of fractures per keel, location (e.g., tip, middle third, dorsal, ventral), fracture type (e.g., transversal, oblique, comminuted, greenstick), direction (e.g., ventral to dorsal), width of the fractures gap, dislocation, or angle between fracture segments, sclerosis, or callus formation were not evaluated as part of the scoring system. However, we assumed that the total amount of bone affected and thus the aggregate fracture severity of an individual hen was contingent on the measures described above, e.g., a comminuted fracture at the tip of the keel would affect less bone than an oblique fracture at the cranial part of the keel. The total amount of bone affected and thus the aggregate fracture severity of an individual hen was determined subjectively using a tagged visual analogue scale (tVAS).

The scoring system consisted of two elements: a tVAS with six visual tags as a scaling tool for a rough classification in a first step and a catalog of “example scores” to refine the tendency to a high or low value between two tags in a second step. The continuous tVAS was a 10 cm line, ranging from score 0 (“no fracture”) to score 5 (“extremely severe”). The scale was tagged at intervals of 2 cm with scores 1, 2, 3, and 4. After marking the line anywhere between score 0 and score 5, the distance from the left anchor of the scale (score 0) was measured with a ruler, resulting in a continuous variable ranging from 0.0 to 10.0 cm (“tVAS range”) or, when divided by two, in a continuous variable ranging from score 0.0 to score 5.0 (“score range”).

**Figure 1:**
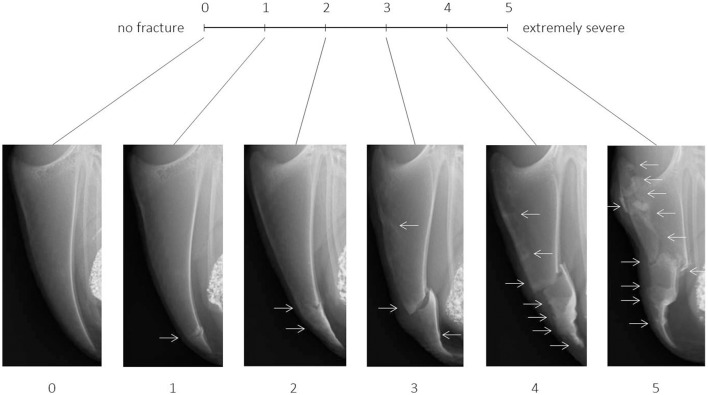
Tagged visual analogue scale ranging from 0 (no fracture) to 5 (extremely severe) with intermediate scores and corresponding example images. Arrows indicate the location of one or multiple fresh, healing, or healed fractures.

For each of the six distinct scores of the tVAS, one radiographic image representing this exact score was added to the scaling tool (Figure 1). As suggested by McCormack (23), the images anchoring the 10 cm line represented the maximal and minimal extreme of the measured dimension: The image for score 0 (left anchor; “no fracture”) showed a fully ossified keel bone of a young hen (22 weeks of age) without fractures or any sign of bone alterations such as sclerosis or increased radiographic density. For score 5 (right anchor; “extremely severe”), the image of the keel bone with the most fractures (*n* = 9) affecting the greatest amount of bone was selected from the total set of 1,622 radiographs. Images representing the intermediate scores 1, 2, 3, and 4 were selected based on intermediate amounts of bone affected by fractures while taking into account the fracture location(s), fracture type(s) and fracture gap properties most frequently observed within the total set of images.

**Figure 2:**
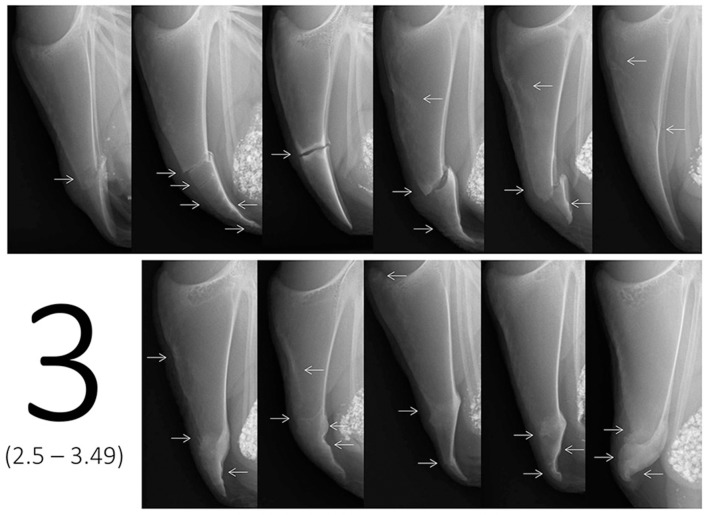
Example images for score 3, i.e., ranging from score 2.5 to score 3.49 on the tagged visual analogue scale. Arrows indicate the location of one or multiple fresh, healing, or healed fractures.

In addition to the scaling tool, a catalog of example scores containing multiple images for each of the six scores was provided in order to visually describe the range of each score. The reason for using multiple representative example images instead of a detailed description or an identification key for scores was the complexity in keels as soon as multiple fractures occurred. Fracture numbers, locations, and characteristics were too diverse to limit all potential cases to one score. Therefore, 10 to 11 example images being similar to the radiographs presented on the scaling tool were assigned to a specific score range taking into account the number of fractures, fracture location(s), fracture type(s), dislocation, angles, and width of the fracture gaps as well as presence of callus material. Example images covered both the most common fracture combinations as well as a few isolated cases as indicated in Figure 2. For instance, most cases resulting in a score 3 would be represented by multiple fractures at the lower part or single fractures with dislocation or wide fracture gaps in the middle part of the keel bone. However, in the example provided (Figure 2), an oblique fracture occurring throughout the upper part of the keel bone would be scored within the range of score 3 as well.

In contrast to the images used for the scaling tool (one image for an exact score), the images of the example scores didn't represent one exact value, but covered the different cases of fractures or combinations that would lie within a specific score range on the tVAS. As an example, Figure 2 shows 11 example radiographs ranging from score 2.5 to 3.49 resulting in a value of 5.0–6.9 cm on the tVAS. After a rough classification using the scaling tool in a first step (e.g., “between 2.5 and 3.49”), the catalog of example images for each of the six scores (i.e., 0, 1, 2, 3, 4, 5) could then be used in a second step to decide on the tendency for either a high or low score between two tags (i.e., “2.7”).

### Online tutorial and reliability trial

In order to test the reliability of the scoring system, we created an online e-learning tool consisting of an introduction, training session, and scoring session. The link for the e-learning tool is open-access and available by contacting the corresponding author or at http://www.keelbonedamage.eu/activities/practical-information-for-stakeholders/online-tool-for-evaluating-fractures-from-radiographic-images/. The web link to the online tutorial was provided to 18 participants of the KeelBoneDamage EU COST Action (http://www.keelbonedamage.eu/) with current or future interest in keel bone fracture assessment. Fourteen people (9 females, 5 males) completed both the training and the scoring session. Participants were based within eight different nations (86% within Europe) and had different educational backgrounds (five veterinarians, four technicians, four scientists, one student; Table 1). All participants had experience with laying hens, but one had no previous experience with keel bones. Twelve out of 14 participants had experience with palpation and/or dissection. Fifty percent of participants were familiar with radiographic assessment in other species, and 57% had experience with radiographs of laying hen keel bones. All participants were asked to read the introduction of the e-learning tool carefully before completing the training session according to the instructions. The subsequent scoring session was only available for the participants of the reliability trial as it aimed to assess inter- and intra-observer reliability, whereas prospective users would only be provided with the introduction and the training session.

**Table 1:** Country, background, and experience of participants of the reliability trial.

**Background**	**Country**	**Experience with…**				
		**Laying hens**	**Keel bones**	**Palpation or dissection**	**Radiographs in general**	**Keel bone radiographs**
Veterinarian	Germany	Yes	Yes	Yes	Yes	Yes
Veterinarian	Egypt	Yes	Yes	Yes	Yes	Yes
Veterinarian	Switzerland	Yes	Yes	Yes	Yes	Yes
Scientist	USA	Yes	Yes	Yes	Yes	Yes
Scientist	Germany	Yes	Yes	Yes	Yes	Yes
Veterinarian	Austria	Yes	Yes	Yes	Yes	No
Technician	Germany	Yes	Yes	Yes	No	Yes
Technician	Germany	Yes	Yes	Yes	No	Yes
Technician	Germany	Yes	Yes	Yes	No	Yes
Scientist	Sweden	Yes	Yes	Yes	No	No
Scientist	Denmark	Yes	Yes	Yes	No	No
Technician	UK	Yes	Yes	Yes	No	No
Student	Switzerland	Yes	Yes	No	No	No
Scientist	Germany	Yes	No	No	Yes	No

The introduction of the e-learning tool gave a background on the detection of fractures using radiographs, explained the aim of both the scoring system and the reliability trial and gave detailed instructions on the use of a tVAS and the example score catalog. All required documents (scaling tool, example score catalog, and empty scales for scoring) were provided as PDF files.

The subsequent training session served to train the user to correctly classify images within an established range. All 65 images used in the example score catalog were presented in a random order. Users had to select the score range (single choice of “score 0,” “score 1,” “score 2,” “score 3,” “score 4,” or “score 5”) of an image using the scaling tool only and received feedback immediately on whether their response was correct. If the image was classified incorrectly, a prompt for another answer was given. Participants were instructed to consult the catalog of example scores after they had selected the correct answer, irrespective of the number of attempts needed. If the image was classified correctly at the first attempt, participants were asked to compare the scored image with the other images of the same score in order to identify the tendency to assign either a high or a low value within the score range. If the image was classified incorrectly, the catalog could be consulted in order to identify why the classification of this specific case was difficult (e.g., unique features, borderline score value).

After completion of the training session, participants of the reliability trial scored 25 images different from those in the example score catalog. Five images per participant were scored twice in order to assess intra-observer reliability. Images were presented on the screen and participants were asked to mark a 10 cm scale on a sheet of paper for each image. For the scoring session, participants could use both the scaling tool and the example score catalog. After completion of the scoring session, participants were asked to scan their scoring sheets and send it to the trial coordinator (CR) as a PDF file. Distance from the left end of the scale (score 0) to the mark was measured with a ruler and entered into a spreadsheet. Total length of the scale was measured as well in order to correct for distortions (scale ≠ 10 cm), e.g., due to different printer settings.

### Statistical analysis

To assess inter-observer reliability, an Intraclass correlation coefficient (ICC) estimate and its 95% confident intervals were calculated using R 3.4.0 (24), package “irr” (25) based on an average-rating (*k* = 14), absolute-agreement, two-way random-effects model (26). To evaluate intra-observer reliability, an ICC estimate and its 95% confident intervals were calculated based on a single-rating, absolute-agreement, two-way mixed-effects model (27, 28). In order to show the range of intra-observer reliability within observers, ICCs were additionally calculated for each observer (*k* = 14) separately. Reliabilities were considered poor (ICC < 0.40), fair (0.40 < ICC < 0.59), good (0.6 < ICC < 0.74), or excellent (0.75 < ICC < 1.0) according to the recommendations of Cicchetti (29).

## Results

### Inter-observer reliability

Intraclass correlation coefficient for inter-observer reliability was 0.985 with a confidence interval of 0.974 < ICC < 0.993 [*F*_(23, 154)_ = 85.7, *p* < 0.0001].

### Intra-observer reliability

Intraclass correlation coefficient for intra-observer reliability was 0.923 with a confidence interval of 0.879 < ICC < 0.951 [*F*_(69, 70)_ = 24.8, *p* < 0.0001]. Individual intra-observer reliability ranged from 0.704 to 1.0.

## Discussion

The aim of this study was to test the reliability of a radiograph scoring system based on a tVAS that allowed for a continuous measure of fracture severity. Both inter- and intra-observer reliability as well as confidence intervals of the estimates were in an excellent range (29), suggesting high agreement across and within participants. High ICCs further indicated minimal measurement errors introduced by the observers (30).

We found excellent reliability even though the use of intermediate tags on a visual analogue scale (VAS) is neither common nor recommended due to likely clustering around the tags (31–33). Other studies investigating welfare issues in farm animals using a VAS with intermediated tags (=tVAS) found fair repeatability (*r* = 0.44) across multiple observers for lameness in cows (34), and an excellent repeatability within the same observer (*r* = 0.98) for feather condition in broiler breeders (35). On the other hand, numerous reports assessing clinical phenomena in human medicine involving sensory or affective states such as pain, mood, anxiety or depression subjectively from a patient's point of view (23, 36) have used VAS without tags successfully. When a VAS without intermediate tags was applied to study measures of animal welfare, inter-observer reliability ranged from fair [ICC = 0.46 (37)] to good [ICC = 0.72 (38)], or excellent [*R*^2^ < 0.82 (39)]. As the current study didn't manifest clustering around the tags and both inter—and intra-observer reliability were in an excellent range, we conclude that intermediate tags in combination with the example score catalog were a beneficial aid to score fracture severity.

Excellent agreement across and within observers in the current study suggest that the e-tutorial provided sufficient background and appropriate training for people with various educational backgrounds and experience. Free access to all materials (scaling tool, example score catalog, background, and training session) would facilitate comparable results between research groups using radiographic imaging for keel bone fracture assessment. Therefore, we believe our radiograph scoring system would be a useful and reliable tool for future studies to aid comparison between and across individual research efforts. As suggested by Casey-Trott et al. (6), we also created a freely accessible online tool (available at http://www.keelbonedamage.eu/ or via the corresponding author) which could be used to recalibrate researchers' scoring skills periodically, e.g., after a long break. The tool would also serve to prevent drift from the initial protocol over time, a common problem in behavioral scoring efforts known as “observer drift” (40).

Unlike palpation, radiographic imaging allows preserving images and enables repeated assessments of the raw data, e.g., with multiple observers or for direct comparison with radiographs from other studies. As radiographic imaging is not practicable within all settings (e.g., for high animal numbers, or due to logistical requirements with equipment), it is unlikely that radiographs will entirely replace palpation for fracture assessment though it would be useful to compare radiograph outcomes with palpation results. However, the detailed information that can be obtained from radiographs and even the simplified measure of aggregate fracture severity are difficult to connect with outcomes from palpation as the variety of existing palpation schemes are often not comparable among themselves. For instance, some systems use a binary outcome [i.e., presence vs. absence (41–43)], whereas others use a four point scale ranging from “no damage” to “severe damage” (44). As palpation will presumably continue to be the most frequently used technique to detect keel bone fractures, we recommend using radiographic imaging as an aid for palpation training in order to enhance accuracy and reliability of keel bone fracture detection using palpation. Direct comparison of palpation outcomes with radiographic images of the same bird will benefit to align the assessor's tactile perception of specific structures with the more exact information about fracture severity which a radiograph could provide. Comparing palpation directly with corresponding radiographs has been used successfully during a training school on keel bone assessment at the University of Bern, where a single day of radiograph-assisted palpation training increased palpation accuracy by 10% (Dr. Sabine Gebhardt-Henrich, personal communication).

Besides being reliable, an index needs to be feasible and valid (45). Feasibility becomes important regarding the execution of radiographic imaging itself due to technical logistics, i.e., equipment, infrastructure, radiation protection, labor, and education are required to conduct radiography. Nevertheless, once the radiographs are produced and an assessor is fully trained to use the scoring system, images could be scored rapidly with 5 to 30 s required per image as with the present study (C. Rufener, personal experience).

To evaluate the validity of our scoring system regarding the effects on animal welfare or other outputs, e.g., productivity, the link between fracture severity and specific measures or relevant indicators should be investigated. Tuyttens et al. (34) suggested comparing a VAS outcome with an independent method or a gold standard. For instance, a VAS for lameness assessment in dogs has been validated by objectively measuring the force distribution on each limb with a force plate and linking these measures with the subjective outcome of the VAS (37, 46).

In our case, no gold standard for aggregate severity assessment of keel bone radiographs exists. Previous radiographic evaluation protocols were only developed for single fractures (i.e., not for entire keel bones) and in a descriptive manner (14, 47) thus complicating the link between the severity of single fractures and individual hen data (e.g., body weight) if multiple fractures occurred. Our scaling tool does not include specific fracture characteristics relevant for clinical examinations (e.g., fracture location), but provides a simplified measure of cumulative damage and therefore aggregate severity of individual hens. While there is evidence that the presence of keel bone fractures has a negative effect on multiple aspects of laying hen welfare [reviewed by Riber et al. (3)], it remains unclear how the severity of fractures affects an individual hen. Thus, as suggested by Harlander-Matauschek et al. (48), our scaling tool must be validated regarding the effect of fracture severity on various animal welfare related indicators (e.g., pain, affective states) as well as other responses of interest (e.g., productivity). As the tVAS provides a continuous measure and because different underlying mechanisms might be involved in response to fractures (e.g., pain leading to reduced mobility; metabolic changes resulting in decreased productivity), linking severity and the magnitude of responses of interest is critical.

## Conclusion

Radiographs of keel bones can provide detailed information on fracture characteristics such as location (e.g., tip, middle third, dorsal, ventral), type (e.g., transversal, comminuted, greenstick), direction (e.g., ventral to dorsal), width of the fractures gap, or callus formation. As laying hens are often affected by multiple fractures, our scoring system aimed to assess aggregate fracture severity of individual hens. Our system compromises on the loss of information regarding specific fracture level characteristics (e.g., fractures at specific locations being more severe than others) for the benefit of a simplified, easy to learn and broadly applicable scoring system. Despite being subjective by definition, the tVAS together with the example score catalog was found to be suitable for observers with different backgrounds and experience after the completion of an online tutorial. Open access to the method and the training protocol, availability of a recalibration tool as well as excellent reliability between and within observers indicated potential for improved comparability among studies using radiographs and the tVAS for fracture assessment. However, further research is needed to validate the scoring system as severity values ascertained with our system need to be linked with relevant measures and indicators describing fields of interest such as pain, behavior, or productivity.

## Ethics statement

Ethical approval to conduct the main study was obtained from the Veterinary Office of the Canton of Bern in Switzerland (approval number BE31/15).

## Author contributions

CR was the principal developer of the scoring system, made the e-tutorial, conducted the reliability trial, analyzed data, and was the principal author of the manuscript. AS assisted in developing the scoring system, refining the e-tutorial, and reviewed the manuscript. SB produced all radiographs, refined the e-tutorial, and gave valuable input. MT initiated the reliability trial, supervised the project, and reviewed the manuscript.

### Conflict of interest statement

The authors declare that the research was conducted in the absence of any commercial or financial relationships that could be construed as a potential conflict of interest.
